# Relationship between neighborhood census-tract level socioeconomic status and respiratory syncytial virus-associated hospitalizations in U.S. adults, 2015–2017

**DOI:** 10.1186/s12879-021-05989-w

**Published:** 2021-03-23

**Authors:** Jenna E. Holmen, Lindsay Kim, Bryanna Cikesh, Pam Daily Kirley, Shua J. Chai, Nancy M. Bennett, Christina B. Felsen, Patricia Ryan, Maya Monroe, Evan J. Anderson, Kyle P. Openo, Kathryn Como-Sabetti, Erica Bye, H. Keipp Talbot, William Schaffner, Alison Muse, Grant R. Barney, Michael Whitaker, Jennifer Ahern, Christopher Rowe, Gayle Langley, Art Reingold

**Affiliations:** 1 UCSF Benioff Children’s Hospital, 747 52nd St, Oakland, CA 94609 USA; 2 Centers for Disease Control and Prevention (CDC), Atlanta, GA USA; 3 US Public Health Service, Atlanta, GA USA; 4 California Emerging Infections Program, Oakland, CA USA; 5 University of Rochester School of Medicine and Dentistry, Rochester, NY USA; 6 Maryland Department of Health, Baltimore, MD USA; 7 Departments of Medicine and Pediatrics, Emory University School of Medicine, Atlanta, GA USA; 8 Emerging Infections Program, Georgia Department of Health, Atlanta, GA USA; 9 Veterans Affairs Medical Center, Atlanta, GA USA; 10 Foundation for Atlanta Veterans Education and Research, Decatur, GA USA; 11 Minnesota Department of Health, St. Paul, MN USA; 12 Vanderbilt University Medical Center, Nashville, TN USA; 13 New York State Department of Health, Albany, NY USA; 14 Division of Epidemiology, School of Public Health, University of California, Berkeley, CA USA; 15 San Francisco Department of Public Health, San Francisco, CA USA

**Keywords:** RSV, Socioeconomic status

## Abstract

**Background:**

Respiratory syncytial virus (RSV) infection causes substantial morbidity and mortality in children and adults. Socioeconomic status (SES) is known to influence many health outcomes, but there have been few studies of the relationship between RSV-associated illness and SES, particularly in adults. Understanding this association is important in order to identify and address disparities and to prioritize resources for prevention.

**Methods:**

Adults hospitalized with a laboratory-confirmed RSV infection were identified through population-based surveillance at multiple sites in the U.S. The incidence of RSV-associated hospitalizations was calculated by census-tract (CT) poverty and crowding, adjusted for age. Log binomial regression was used to evaluate the association between Intensive Care Unit (ICU) admission or death and CT poverty and crowding.

**Results:**

Among the 1713 cases, RSV-associated hospitalization correlated with increased CT level poverty and crowding. The incidence rate of RSV-associated hospitalization was 2.58 (CI 2.23, 2.98) times higher in CTs with the highest as compared to the lowest percentages of individuals living below the poverty level (≥ 20 and < 5%, respectively). The incidence rate of RSV-associated hospitalization was 1.52 (CI 1.33, 1.73) times higher in CTs with the highest as compared to the lowest levels of crowding (≥5 and < 1% of households with > 1 occupant/room, respectively). Neither CT level poverty nor crowding had a correlation with ICU admission or death.

**Conclusions:**

Poverty and crowding at CT level were associated with increased incidence of RSV-associated hospitalization, but not with more severe RSV disease. Efforts to reduce the incidence of RSV disease should consider SES.

**Supplementary Information:**

The online version contains supplementary material available at 10.1186/s12879-021-05989-w.

## Key points

Higher census-tract level poverty and crowding are associated with increased incidence of RSV-associated hospitalization. Census-tract level poverty and crowding did not correlate with either ICU admission or in-hospital mortality.

## Background

Respiratory syncytial virus (RSV) is an established cause of morbidity and mortality in infants and children, and its importance in adults is being increasingly recognized. In the U.S. adult population, RSV accounts for roughly 12% of medically-attended respiratory illnesses with a case fatality proportion of 6–8% in those over 50 years of age [1]. Overall, RSV infection is associated with an average of 16,272–18,444 deaths (adults and children) per year in the U.S. [2] Other data suggest that 78% of RSV-associated deaths occur in adults > 65 years of age [3]. In addition to older age being a risk factor for more severe RSV disease, adults with comorbid conditions, particularly chronic cardiopulmonary disorders, are particularly vulnerable to severe RSV-associated illness [4–9].

Factors related to socioeconomic status (SES) play an important role in an individual’s health and disease status [10, 11]. Evaluating the relationship between illness and SES at the census-tract level can be particularly valuable, because an individual’s health is influenced by a constellation of variables at both the individual and the neighborhood-levels [12–16]. Census-tracts are small, relatively permanent geographic subdivisions that are standardized and constructed to contain approximately 4000 individuals of relatively similar population characteristics and economic status [17]. Further, working with surveillance data offers a way to examine patterns of incidence by SES, using measures not typically available for individual cases. Census-tract level poverty has been found to be one of the most sensitive and consistent metrics by which to assess the association between disease and area/neighborhood socioeconomic status [12, 14, 15].

Studies of both adults and children evaluating the incidence of influenza in relationship to census-tract level SES factors have shown a disproportionately higher incidence of influenza-associated hospitalizations in individuals living in census-tracts with higher levels of poverty and crowding [18–20]. The relationship between RSV infection and SES has not been well studied, though several previous studies suggest an association between the incidence of RSV infection and lower SES [21–25]. However, there have not yet been any national studies evaluating the association of RSV-associated hospitalizations and SES at the census-tract level.

In this study, data from the Centers for Disease Control and Prevention (CDC)‘s Emerging Infections Program (EIP) RSV-associated hospitalization population-based surveillance (RSV-NET) were analyzed to evaluate whether the incidence of RSV-associated hospitalizations was associated with census-tract level poverty and crowding. Further analysis evaluated whether RSV disease severity (as defined by in-hospital death or ICU admission) was associated with census-tract level poverty and crowding among those hospitalized with RSV.

## Methods

### Overview of surveillance system, study population, and data collection

The EIP is a network of state health departments and their collaborators in local health departments, public health laboratories, and clinical laboratories; infection control professionals; healthcare providers; and academic institutions. This network provides a national resource for infectious disease surveillance and response, conducts applied epidemiologic and laboratory research, identifies measures to prevent and control emerging infectious diseases, and strengthens national public health infrastructure [26]. RSV surveillance began during the 2016–2017 respiratory season (October 1–April 30) with retrospective surveillance for the 2014–2015 and 2015–2016 respiratory seasons in 6 states (California, Georgia, Minnesota, New York, Oregon, and Tennessee). Maryland was added in 2017–18. A case is defined as an adult ≥18 years that is a resident of the catchment area admitted ≤14 days after a laboratory-confirmed RSV test during the respiratory season to a hospital where catchment area residents receive care. Hospitalization is defined as admission to an inpatient ward of the hospital or a stay in an observation unit that is ≥24 h or a combined stay in an emergency department and observation unit for ≥24 h. Patients who are seen only in the Emergency Department or outpatient clinic (even if for more than 24 h) are not cases. Patients who are admitted to and discharged from the hospital on the same day are considered hospitalized (i.e. an overnight stay is not required). A patient was considered to have an RSV infection if RSV was detected by: rapid antigen, molecular assay, viral culture, or fluorescent antibody. Cases were identified using hospital and state public health laboratory databases, hospital infection control practitioner (ICP) databases/logs, reportable condition databases, electronic medical records (EMR), and/or review of hospital discharge records. Trained surveillance officers completed medical record abstraction using a standardized case report form.

For this analysis, cases from 33 counties in California, Georgia, Maryland, Minnesota, New York, and Tennessee were included. Oregon was excluded due to lack of geocoding data. Demographic and clinical variables collected included patient age, sex, race, ethnicity, residential street address; the presence of comorbid conditions; any substance use; and clinical course including ICU admission and in-hospital death. Participants’ addresses were geocoded using ArcGIS software.

### Census data

The percentage of the population living in poverty and crowding was calculated at the census-tract level using data from the most recent five-year (2013–2017) American Community Survey (ACS). Census tracts were then divided into four levels of poverty by percentage of people living below the poverty level: 0–4.9%, 5–9.9%, 10–19.9%, and ≥ 20%. These categories were used per the recommendations of the Public Health Disparities Geocoding Project [14, 15]. Data on crowding were extracted for the relevant census tracts by the percentage of households with > 1 occupant/room. This was divided into four levels of crowding by percentage of households within a given census-tract with > 1 occupant per room as follows: low = 0–0.9%, medium low = 1–2.9%, medium high = 3–4.9%, high = ≥5%. Levels were selected as per previous studies [18, 19]. The 2010 US census was used to determine the denominator data of population of each census tract.

### Statistical analysis

For calculation of incidence rates, numerators were obtained from the surveillance data by matching each patient’s geocoded address to their corresponding census-tract. Denominator data were taken from the US census 2010. Patients were divided into four age categories: 18 to < 50 years old, 50 to < 65 years old, 65 to < 80 years old, and ≥ 80 years old and age-adjusted incidence rates were calculated using these age groups and the US census 2010 data as the standard population. A Cuzick test for trend across ordered groups was conducted to compare incidence rates. Incidence rate ratios were calculated comparing each level of poverty/crowding to the lowest level of the respective category.

Log binomial regression models were used to examine the association between census-tract level poverty and crowding with severe RSV-associated illness, defined as either in-hospital death or ICU admission. First, separate unadjusted models were fit with independent variables considered to be potentially associated with severe RSV disease: age, sex, race, ethnicity, obesity as defined as body mass index (BMI) ≥30 kg/m^2^, smoking (current and/or former), presence of an immunocompromised condition (i.e. HIV, immunosuppressive therapy use, primary immune deficiency, graft versus host disease, solid organ transplant, bone marrow transplant, and cancer diagnosis), chronic lung disease (i.e. chronic bronchitis, chronic obstructive pulmonary disease/emphysema, asthma/reactive airway disease and cystic fibrosis), cardiovascular disease (i.e. coronary artery disease, congestive heart failure, cardiomyopathy, history of coronary artery bypass grafting, cerebral vascular accident/stroke/transient ischemic attack, atherosclerosis), and surveillance site. The medical conditions included have previously been identified as possibly being associated with an increased risk of RSV infection or severity of RSV, or other respiratory viral infection [4–9]. Variables that had a statistical association with death or ICU admission at a *p*-value < 0.2 (i.e., age, obesity, and study site) were included along with either census-tract level poverty or crowding in the multivariate model. In the final multivariate model, variables that had an association with death or ICU admission at a p-value of < 0.05 were considered statistically significant. Analyses were conducted using STATA, version 15.1.

## Results

A total of 1713 cases were included in the final analysis, after excluding 45 (2.5%) with missing address information. There were more RSV-associated hospitalizations in adults older than 50 years compared with those less than 50 years of age (Table 1), and there were more women (59%) with RSV-associated hospitalizations. Among adults with an RSV-associated hospitalization, there were more individuals who identified as Black, compared to White, living in census tracts with higher percentages of poverty. Of those with insurance information available, half (50%) had more than one insurance type; the majority (57%) had Medicare insurance, a federally-funded insurance program for adults over 65 years of age and for certain qualifying individuals receiving disability benefits. The proportion of individuals with Medicaid, a federal and state insurance program for people with limited income, increased with increasing census-tract level poverty (Table 1). A total of 94% of patients had at least one comorbid condition, with pulmonary disease (51%) being the most common followed by obesity (48%) and chronic metabolic disease (46%). Smoking was the most common type of current substance use (17%) as compared to alcohol use (3%) and other drugs (3%). There was a greater percentage of current smokers (29%) in census tracts with the highest levels of poverty compared to the census tracts with the lowest levels of poverty (10%). There were 86 (5%) total in-hospital deaths among hospitalized cases. The percentage of in-hospital deaths by census-tract poverty level population (5%) was distributed evenly across each level of census-tract poverty (Table 2).

**Table 1:** Demographic characteristics of adults with an RSV-associated hospitalization, 2015–2017

Demographic characteristics (1713 cases), n (%)					
	Percent of individuals living below the poverty level within a census tract				Total (***n*** = 1713)
Patient age (years)	**0–4.9%** **(*n*** **= 397)**	**5–9.9%** **(*n*** **= 535)**	**10–19.9%** **(*n*** **= 438)**	**≥20%** **(*n*** **= 343)**	
18- < 49	49 (12)	59 (11)	70 (16)	73 (21)	251 (15)
50–64	76 (19)	132 (25)	109 (25)	115 (34)	432 (25)
65–79	137 (35)	167 (31)	123 (28)	112 (33)	539 (31)
> 80	135 (34)	177 (33)	136 (31)	43 (13)	491 (29)
Sex					
Male	180 (45)	196 (37)	183 (42)	141 (41)	700 (41)
Female	217 (55)	339 (63)	255 (58)	202 (59)	1013 (59)
Race/Ethnicity					
White	306 (77)	358 (67)	247 (56)	123 (36)	1034 (60)
Black	34 (9)	78 (15)	116 (26)	171 (50)	399 (23)
Asian/Pacific Islander	33 (8)	58 (11)	38 (9)	13 (4)	142 (8)
Other/not-reported	4 (1)	5 (1)	3 (1)	3 (1)	15 (1)
Hispanic	15 (4)	31 (6)	25 (6)	30 (9)	101 (6)
Insurance type					
Medicare	234 (59)	321 (60)	248 (57)	176 (51)	979 (57)
Medicaid	48 (12)	81 (15)	95 (22)	153 (45)	377 (22)
Private insurance	229 (58)	248 (46)	188 (43)	115 (34)	780 (46)
Uninsured	5 (1)	2 (0)	10 (2)	5 (1)	22 (1)
Other/not-reported	11 (3)	8 (1)	9 (2)	5 (1)	33 (2)
State					
California	123 (31)	199 (37)	143 (33)	57 (17)	522 (30)
Georgia	36 (9)	39 (7)	66 (15)	45 (13)	186 (11)
Maryland	94 (24)	100 (19)	86 (20)	61 (18)	341 (20)
Minnesota	43 (11)	45 (8)	28 (6)	21 (6)	137 (8)
New York	89 (22)	101 (19)	81 (18)	124 (36)	395 (23)
Tennessee	12 (3)	51 (10)	34 (8)	35 (10)	132 (8)

**Table 2:** Clinical characteristics of adults with RSV-associated hospitalization, 2015–2017

Clinical characteristics (number of patients), n (%)					
	Percent of individuals living below the poverty level within a census tract				Total (n = 1713)
Underlying conditions	**0–4.9%** **(n = 397)**	**5–9.9%** **(n = 535)**	**10–19.9%** **(n = 438)**	**≥20%** **(n = 343)**	
Any underlying medical condition	377 (95)	508 (95)	409 (93)	321 (94)	1615 (94)
Pulmonary disease^1^	166 (42)	279 (52)	211 (39)	215 (63)	871 (51)
Obesity (BMI ≥30)	203 (51)	244 (46)	212 (48)	158 (46)	817 (48)
Chronic metabolic disease^2^	171 (43)	269 (32)	189 (35)	163 (48)	792 (46)
Cardiovascular disease^3^	170 (43)	245 (46)	182 (42)	156 (45)	753 (44)
Renal disease^4^	95 (24)	163 (30)	126 (29)	106 (31)	490 (29)
Immunocompromised^5^	104 (26)	136 (25)	89 (17)	93 (27)	422 (25)
Neurologic disease^6^	87 (22)	105 (20)	91 (17)	64 (19)	347 (20)
Liver disease^7^	20 (5)	29 (7)	22 (5)	30 (9)	101 (6)
Blood disorders/hemoglobinopathy^8^	16 (4)	17 (3)	16 (3)	10 (3)	59 (3)
Rheumatologic disease ^9^	18 (5)	17 (3)	15 (3)	22 (6)	72 (4)
Substance Use					
Smoker (current)	39 (10)	64 (12)	88 (20)	101 (29)	292 (17)
Smoker (former)	153 (39)	178 (33)	131 (30)	105 (31)	567 (33)
Smoker (no/unknown)	205 (52)	293 (55)	219 (50)	137 (40)	854 (50)
Alcohol abuse (current)	8 (2)	11 (2)	14 (3)	25 (7)	58 (3)
Alcohol abuse (former)	13 (3)	16 (3)	10 (2)	13 (4)	52 (3)
Alcohol abuse (no/unknown)	376 (95)	508 (95)	414 (95)	305 (89)	1603 (94)
Substance abuse (current)	5 (1)	8 (1)	10 (2)	28 (8)	51 (3)
Substance abuse (former)	2 (1)	3 (1)	2 (0)	9 (3)	16 (1)
Substance abuse (no/unknown)	390 (98)	524 (98)	426 (98)	306 (89)	1646 (96)
Indicators of Severe Illness					
Hospital stay > 7 days	103 (26)	110 (21)	106 (24)	103 (30)	422 (25)
ICU admission	86 (22)	92 (17)	86 (20)	80 (23)	344 (20)
Need for mechanical ventilation	32 (8)	39 (7)	35 (8)	37 (11)	143 (8)
Death	19 (5)	28 (5)	21 (5)	18 (5)	86 (5)

^1^asthma, COPD, chronic bronchitis, emphysema, CF, Chronic respiratory failure, active TB, other

^2^diabetes, thyroid disease, other endocrinopathy

^3^coronary artery disease, congestive heart failure, cardiomyopathy, prior coronary artery bypass graft, cerebral vascular accident/stroke/transient ischemic attack, atherosclerosis

^4^chronic kidney disease, end-stage renal disease, glomerulonephritis, nephrotic syndrome, other

^5^cancer, complement deficiency, HIV, AIDS, immunoglobulin deficiency, any immunosuppressive therapy, status-post solid organ transplant, s/p bone marrow transplant, receipt of steroid therapy, other

^6^cerebral palsy, cognitive dysfunction, dementia, developmental delay, Trisomy 21, paralysis, seizures, muscular dystrophy, multiple sclerosis, myasthenia gravis, other

^7^cirrhosis, hepatitis B or C, other

^8^aplastic anemia, Sickle cell disease, asplenia, other

^9^lupus, Juvenile idiopathic arthritis, rheumatoid arthritis, psoriasis, polymyositis, polymyalgia, polyarteritis nodosa, vasculitis, other

**Fig. 1:**
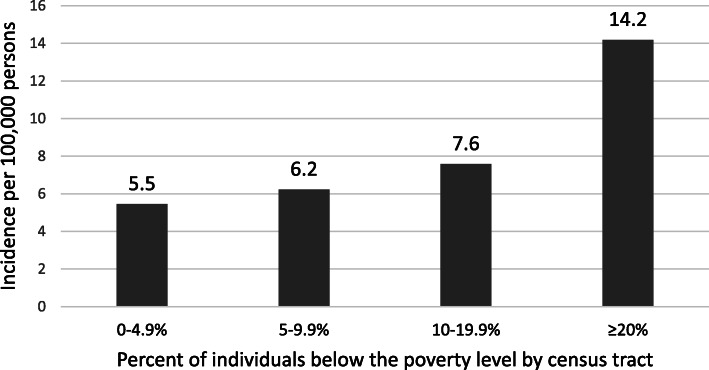
Age-adjusted incidence rate of RSV-associated hospitalizations of adults by census-tract poverty level, 2015–2017. Test for trend *p*-value: < 0.005

**Fig. 2:**
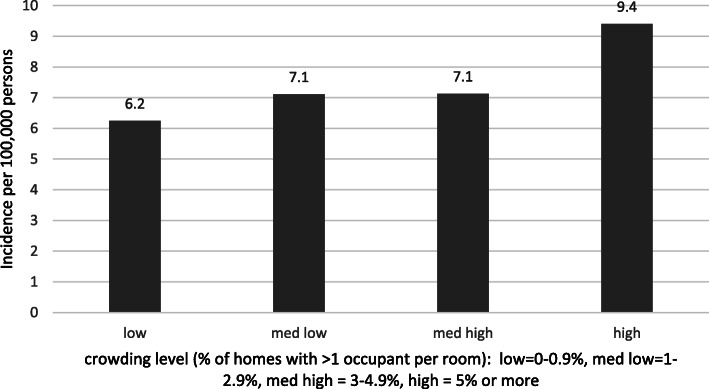
Age-adjusted incidence rate of RSV-associated hospitalization in adults by census-tract crowding, 2015–2017. Test for trend *p*-value: < 0.005

The incidence of RSV-associated hospitalizations of adults increased with higher levels of census-tract poverty (Fig. 1), with a statistically significant test for trend (*p* < 0.005). In census tracts with the highest percentages of individuals living in poverty, the rate of RSV-associated hospitalizations was 2.58 (CI 2.23–2.98) times the rate among those living in census tracts with the lowest percentage of individuals living in poverty (Table 3). The incidence of RSV-associated hospitalizations among adults increased with higher levels of census-tract level crowding (Fig. 2), with a statistically significant test for trend (p < 0.005). The rate of RSV-associated hospitalization among individuals living in neighborhoods with the highest percentages of crowding was 1.52 (CI 1.33–1.73) times higher than that in individuals living in neighborhoods with the lowest levels of crowding (Table 4).

**Table 3:** Incidence rate ratios for RSV-associated hospitalizations of adults by census-tract poverty level, 2015–2017

Poverty levels (all sites)	Incidence rate ratio	95% Confidence Interval
0–4.9%	–	–
5–9.9%	1.12	0.98, 1.28
10–19.9%	1.38	1.20, 1.58
≥20%	2.58	2.23, 2.98

**Table 4:** Incidence rate ratios for RSV-associated hospitalization in adults by census-tract crowding, 2015–2017

Neighborhood crowding	Incidence rate ratio	95% Confidence Interval
Low	–	–
Medium low	1.14	1.01–1.28
Medium high	1.14	0.98–1.33
High	1.52	1.33, 1.73

Neither census-tract level poverty nor crowding had an association with either ICU admission or death in either bivariate or multivariate analysis (See Additional Files 1 and 2).

## Discussion

In this study, higher census-tract level poverty was associated with a higher incidence of RSV-associated hospitalization in adults. This association is likely multifactorial, with poverty unlikely being the sole explanatory component. Higher census-tract crowding was also associated with a higher incidence of RSV-associated hospitalizations, particularly when comparing neighborhoods with the lowest levels of crowding with neighborhoods with the highest levels of crowding.

This study was conducted using census-tract level data because individual metrics of SES are limited in surveillance data and because neighborhood-level variables have an impact on a person’s health, including healthcare-related variables such as access to care; infrastructure variables, such as sanitation, neighborhood density, and access to resources; and environmental variables, such as pollution [27]. All of these variables can be associated with SES and affect a person’s health and are best studied on the census-tract level [12–14]. Furthermore, among census-tract variables associated with SES, census-tract level poverty has been shown to be the most sensitive and consistent metric by which to assess the association between disease and SES [12, 14, 15]. The finding of increasing incidence of RSV-associated hospitalization among individuals living in census-tracts with increasing percentages of residents living below poverty most likely reflects the sum of the negative health impacts of social determinants of health associated with living in impoverished communities.

The influence of poverty on RSV incidence and severity in adults living in low income countries is likely to be even more pronounced. While data on RSV incidence is limited in adults, studies looking at RSV lower respiratory tract disease in children have shown an incidence of RSV-associated lower respiratory tract disease over two times higher in developing as compared to industrialized countries, as well as an RSV-associated case fatality ratio 3–7 times higher in developing as compared to industrialized countries [28]. In low income countries where there is less access to medical care and other resources, the association between poverty and RSV incidence and mortality is likely to be more robust in both children and adults.

These results are consistent with those of other studies comparing disease incidence among the highest and lowest categories of census-tract level poverty, as well as census tract-level crowding [14, 15]. Interestingly, incident rates were higher in census-tracts with high levels of poverty than in census tracts with high levels of crowding, which is different from the findings of prior studies that have found the opposite pattern [14, 15]. These prior studies examined the incidence of other diseases, including sexually transmitted infections (STIs) and tuberculosis (TB) [14, 15]. RSV infection differs from STI and TB in that a prior infection with RSV can provide partial immunity [29–31]. Prior studies have shown that the risk of RSV infection is inversely related to the level of neutralizing antibodies and that repeat infections with RSV tend to be more mild [32, 33]. It is plausible that individuals living in more crowded neighborhoods have frequent and repeated infections with RSV and thus may be partially protected from disease severe enough to warrant hospitalization. This partial protection from severe disease may explain why census-tract level crowding was a less robust marker of disparities related to RSV-associated hospitalization as opposed to census-tract level poverty.

The findings in this study are similar to those from prior studies of influenza. Studies of influenza using census-tract data have shown an increasing incidence of adults with influenza-associated hospitalization corresponding to increasing census-tract level poverty, as well as with census-tract level crowding [18]. Similar to these findings, studies of influenza have also failed to find an association between severe disease, defined as death or ICU admission, and census-tract level poverty [34].

Recognizing and eliminating health disparities is an important goal of the CDC’s Healthy People 2020 [10, 11]. Identifying and quantifying health disparities is essential to establish a baseline measure from which to evaluate the impact of future interventions aimed at eliminating these disparities. Understanding SES-related disparities associated with RSV infection is critical, given that there are promising vaccines on the horizon [35]. There are currently a number of RSV vaccines in development, including several targeting the elderly, that are in phase 2 and 3 trials [35]. It is important to understand the epidemiologic features of RSV disease to provide a baseline against which to measure the impact of future intervention efforts on health disparities and to guide future vaccine recommendations.

This study was limited by its inclusion of only hospitalized, laboratory-confirmed cases. Cases are thus not reflective of the overall burden of RSV disease, which is most commonly managed on an outpatient basis [36, 37]. Furthermore, identification of RSV-associated hospitalized cases through RSV-NET is dependent on clinician testing; limited numbers of patients undergo RSV testing, even among the subset of patients who are admitted to the hospital [38]. There can also be biases in the decision to admit patients and to test for RSV both within and between different institutions that could have influenced the results. Because only in-hospital deaths are captured, our severity analysis may have been influenced by the higher hospitalization rate among individuals living in higher poverty as we don’t know how many individuals from lower poverty census-tracts may have died outside the hospital.

## Conclusions

In conclusion, an association was found between the incidence of RSV-associated hospitalizations in adults and higher census tract-level poverty and crowding, although no relationship was observed between severity as measured by ICU admission and in-hospital mortality with census-tract level poverty or crowding. Future efforts to decrease the burden of RSV disease, including vaccination, should address these disparities. This paper provides a baseline of the incidence of RSV-associated hospitalizations in adults by census-tract SES, by which the impact on these SES disparities of future efforts to decrease the burden of RSV disease can be measured.

## Supplementary Information

**Additional file 1.** Bivariate association of demographic and clinical characteristics with severe RSV disease (death or ICU admission) among patients hospitalized with RSV.

**Additional file 2.** Multivariate analysis of odds of severe RSV disease (death or ICU admission) among adults with RSV-associated hospitalization by poverty category, adjusted for age, obesity, and study site. (Data from Tennessee and Georgia were collinear, so data from Tennessee were omitted)

## Data Availability

The data that support the findings of this study are available from the CDC but restrictions apply to the availability of these data, which were used under license for the current study, and so are not publicly available. Data are however available from the authors upon reasonable request and with permission of the CDC.

## Abbreviations

RSV: Respiratory Syncytial Virus
SES: Socioeconomic Status
CT: census-tract
ICU: Intensive Care Unit
CDC: Centers for Disease Control and Prevention
EIP: Emerging Infections Program
ACS: American Community Survey
STI: Sexually transmitted infection
TB: Tuberculosis
