# Supplementary material for: Relationship between neighborhood census-tract level socioeconomic status and respiratory syncytial virus-associated hospitalizations in U.S. adults, 2015–2017
Source: BMC Infect Dis. 2021 Mar 23;21:293. doi: 10.1186/s12879-021-05989-w (PMC7986301; doi:10.1186/s12879-021-05989-w)
Supplement: Supplementary file 1 — Additional file 1. Bivariate association of demographic and clinical characteristics with severe RSV disease (death or ICU admission) among patients hospitalized with RSV. [file 12879_2021_5989_MOESM1_ESM.docx]

| **Characteristic** | **Odds ratio** | **Confidence Interval** | **p value** |
| --- | --- | --- | --- |
| **Age** | -0.70 | -0.16, 0.01 | 0.11 |
| **Sex** | 0.03 | -0.15, 0.22 | 0.68 |
|  |  |  |  |
| **Black** | -0.11 | -0.33, 0.11 | 0.35 |
| **White** | 0.08 | -0.10, 0.26 | 0.41 |
| **Hispanic Ethnicity** | 0.04 | -0.32, 0.41 | 0.83 |
|  |  |  |  |
| **Smoker (former or current)** | 0.05 | -0.13, 0.23 | 0.56 |
| **Obese (BMI ≥30)** | -0.21 | -0.44, 0.02 | 0.07 |
| **Pulmonary disease** | 0.08 | -0.10, 0.26 | 0.39 |
| **Cardiovascular disease** | 0.13 | -0.05, 0.31 | 0.16 |
| **Immunosuppressed** | 4.3 x 10^-4^ | -1.7 x 10^-3^, 2.5 x10^-3^ | 0.70 |
|  |  |  |  |
| **California** | --- | --- | --- |
| **Georgia** | 0.46 | 0.17, 0.74 | 0.002 |
| **Maryland** | 0.26 | -0.01, 0.52 | 0.06 |
| **Minnesota** | 0.36 | 0.02, 0.69 | 0.04 |
| **New York** | -0.05 | -0.33, 0.23 | 0.72 |
| **Tennessee** | 0.42 | 0.09, 0.75 | 0.01 |
|  |  |  |  |
| **% of individuals living in poverty** |  |  |  |
| **0-4.9%** | --- | --- | --- |
| **5-9.9%** | -0.22 | -0.47, 0.03 | 0.09 |
| **10-19.9%** | -0.12 | -0.38, 0.14 | 0.36 |
| **≥20%** | 0.09 | -0.16, 0.34 | 0.48 |
|  |  |  |  |
| **% of individuals living in crowded neighborhoods (>1 occupant/room)** |  |  |  |
| **0-0.9%** | --- | --- | --- |
| **1-2.9%** | 0.03 | -0.19, 0.25 | 0.80 |
| **3-4.9%** | -0.03 | -0.32, 0.26 | 0.84 |
| **≥5%** | -0.02 | -0.26, 0.23 | 0.90 |

Additional File 1. Bivariate association of demographic and clinical characteristics with severe RSV disease (death or ICU admission) among patients hospitalized with RSV.
