# Supplementary material for: Relationship between neighborhood census-tract level socioeconomic status and respiratory syncytial virus-associated hospitalizations in U.S. adults, 2015–2017
Source: BMC Infect Dis. 2021 Mar 23;21:293. doi: 10.1186/s12879-021-05989-w (PMC7986301; doi:10.1186/s12879-021-05989-w)
Supplement: Supplementary file 2 — Additional file 2. Multivariate analysis of odds of severe RSV disease (death or ICU admission) among adults with RSV-associated hospitalization by poverty category, adjusted for age, obesity, and study site. (Data from Tennessee and Georgia were collinear, so data from Tennessee were omitted) [file 12879_2021_5989_MOESM2_ESM.docx]

| **Variable** | **Odds ratio** | **Confidence interval** | **p value** |
| --- | --- | --- | --- |
| **% of individuals living in poverty** |  |  |  |
| **0-4.9%** | --- | --- | --- |
| **5-9.9%** | -0.19 | -0.47, 0.10 | 0.20 |
| **10-19.9%** | -0.19 | -0.49, 0.10 | 0.20 |
| **≥20%** | -0.01 | -0.32, 0.29 | 0.94 |
|  |  |  |  |
| **Age** | -0.09 | -0.19, 0.01 | 0.09 |
| **Obese (BMI ≥30)** | -0.23 | -0.45, -8.4 x10^-5^ | 0.05 |
|  |  |  |  |
| **California** | --- | --- | --- |
| **Georgia** | 0.33 | 0.12, 0.65 | 0.04 |
| **Maryland** | 0.13 | -0.16, 0.42 | 0.40 |
| **Minnesota** | 0.13 | -0.31, 0.56 | 0.57 |
| **New York** | -0.27 | -0.60, 0.06 | 0.10 |
| **Tennessee** | --- | --- | --- |

Additional File 2a. Multivariate analysis of odds of severe RSV disease (death or ICU admission) among adults with RSV-associated hospitalization by poverty category, adjusted for age, obesity, and study site. (Data from Tennessee and Georgia were collinear, so data from Tennessee were omitted)

| **Variable** | **Odds ratio** | **Confidence interval** | **p value** |
| --- | --- | --- | --- |
| **% of individuals living in crowded neighborhoods (>1 occupant/room)** |  |  |  |
| **0-0.9%** | --- | --- | --- |
| **1-2.9%** | 0.04 | -0.22, 0.30 | 0.79 |
| **3-4.9%** | -0.06 | -0.41, 0.29 | 0.73 |
| **≥5%** | 0.16 | -0.14, 0.47 | 0.30 |
|  |  |  |  |
| **Age** | -0.10 | -0.20, 0.01 | 0.07 |
| **Obese (BMI ≥30)** | -0.24 | -0.46, -0.01 | 0.04 |
|  |  |  |  |
| **California** | --- | --- | --- |
| **Georgia** | 0.41 | 0.08, 0.74 | 0.02 |
| **Maryland** | 0.20 | -0.11, 0.51 | 0.21 |
| **Minnesota** | 0.19 | -0.25, 0.64 | 0.39 |
| **New York** | -0.16 | -0.50, 0.19 | 0.38 |
| **Tennessee** | --- | --- | --- |

Additional File 2b. Multivariate analysis of odds of severe RSV disease (death or ICU admission) among adults with RSV-associated hospitalization by census tract crowding, adjusted for age, obesity, and study site. (Data from Tennessee and Georgia were collinear, so data from Tennessee were omitted)
